# Factors to improve distress and fatigue in Cancer survivorship; further understanding through text analysis of interviews by machine learning

**DOI:** 10.1186/s12885-021-08438-8

**Published:** 2021-06-27

**Authors:** Kyungmi Yang, Jina Kim, Mison Chun, Mi Sun Ahn, Eunae Chon, Jinju Park, Mijin Jung

**Affiliations:** 1 Department of Radiation Oncology, Samsung Medical Center, Sungkyunkwan University School of Medicine, Seoul, Republic of Korea; 2 Department of Radiation Oncology, Ajou University School of Medicine, Suwon, Republic of Korea; 3 Cancer Survivorship Center, Ajou University School of Medicine, Suwon, Republic of Korea; 4 Department of Hematology Oncology, Ajou University School of Medicine, Suwon, Republic of Korea

**Keywords:** Cancer survivorship, Distress, Fatigue, Machine learning, Text analysis

## Abstract

**Background:**

From patient-reported surveys and individual interviews by health care providers, we attempted to identify the significant factors related to the improvement of distress and fatigue for cancer survivors by text analysis with machine learning techniques, as the secondary analysis using the single institute data from the Korean Cancer Survivorship Center Pilot Project.

**Methods:**

Surveys and in-depth interviews from 322 cancer survivors were analyzed to identify their needs and concerns. Among the keywords in the surveys, including EQ-VAS, distress, fatigue, pain, insomnia, anxiety, and depression, distress and fatigue were focused. The interview transcripts were analyzed via Korean-based text analysis with machine learning techniques, based on the keywords used in the survey. Words were generated as vectors and similarity scores were calculated by the distance related to the text’s keywords and frequency. The keywords and selected high-ranked ten words for each keyword based on the similarity were then taken to draw a network map.

**Results:**

Most participants were otherwise healthy females younger than 50 years suffering breast cancer who completed treatment less than 6 months ago. As the 1-month follow-up survey’s results, the improved patients were 56.5 and 58.4% in distress and fatigue scores, respectively. For the improvement of distress, dyspepsia (*p* = 0.006) and initial scores of distress, fatigue, anxiety, and depression (*p* < 0.001, < 0.001, 0.043, and 0.013, respectively) were significantly related. For the improvement of fatigue, economic state (*p* = 0.021), needs for rehabilitation (*p* = 0.035), initial score of fatigue (*p* < 0.001), any intervention (*p* = 0.017), and participation in family care program (*p* = 0.022) were significant. For the text analysis, Stress and Fatigue were placed at the center of the keyword network map, and words were intricately connected. From the regression anlysis combined survey scores and the quantitative variables from the text analysis, participation in family care programs and mention of family-related words were associated with the fatigue improvement (*p* = 0.033).

**Conclusion:**

Common symptoms and practical issues were related to distress and fatigue in the survey. Through text analysis, however, we realized that the specific issues and their relationship such as family problem were more complicated. Although further research needs to explore the hidden problem in cancer patients, this study was meaningful to use personalized approach such as interviews.

**Supplementary Information:**

The online version contains supplementary material available at 10.1186/s12885-021-08438-8.

## Background

In Korea, the number of cancer patients has increased gradually over the years [1]. However, due to the development of therapeutic options, cancer mortality rates have decreased since the early 2000s. Consequently, the number of patients who survive after cancer treatment (cancer survivors) has continuously increased. In Korea, patients diagnosed with cancer between 2013 and 2017 showed a 5-year relative survival rate of 70.4%, which lead to a prevalence of approximately 1.87 million cases at the end of 2017. Although the population of cancer survivors is substantial, the importance of care for cancer survivors is underestimated.

After cancer treatment, the cancer survivors primarily screened for treatment outcomes, including physical complications. However, previous reports described that even after finishing cancer treatment, cancer survivors still experience various issues in physical, mental, and even practical aspects of life [2–5]. Although distress and fatigue are included in the guidelines for cancer survivorship, many survivors have often reported distress and fatigue regardless of disease progression [6]. In most cancer survivorship studies, analyses are based on simple surveys or reports from patients, which may be insufficient for detailed analysis to suggest solutions. Moreover, although many hospitals and public organizations show an interest in cancer survivorship [7], it has practical limitations on budget and resources to provide appropriate care for cancer survivors individually.

This study aimed to find specific and individualized causes behind cancer survivors’ distress and fatigue in existing survey and interview data of the survivorship program through the newly approached machine learning technique for text analysis of the Korean language.

## Methods

The manuscript of this study was prepared according to Strengthening the Reporting of Observationally Studies in Epidemiology (STROBE) guidelines [8].

### Korean Cancer survivorship pilot project

The Korean Cancer Survivorship Pilot Project was launched in 2017 across Regional Cancer Centers in Korea. Adult cancer survivors who completed active cancer treatment such as surgery, radiotherapy, or chemotherapy, excluding palliative care, were recruited for a prospective and observational cohort study approved by IRB (AJIRB-MED-MDB-17-008). One of the regional cancer centers covering Kyung-gi province near Seoul, our institution joined the pilot project as one of the participating regional cancer centers.

**Fig. 1:**
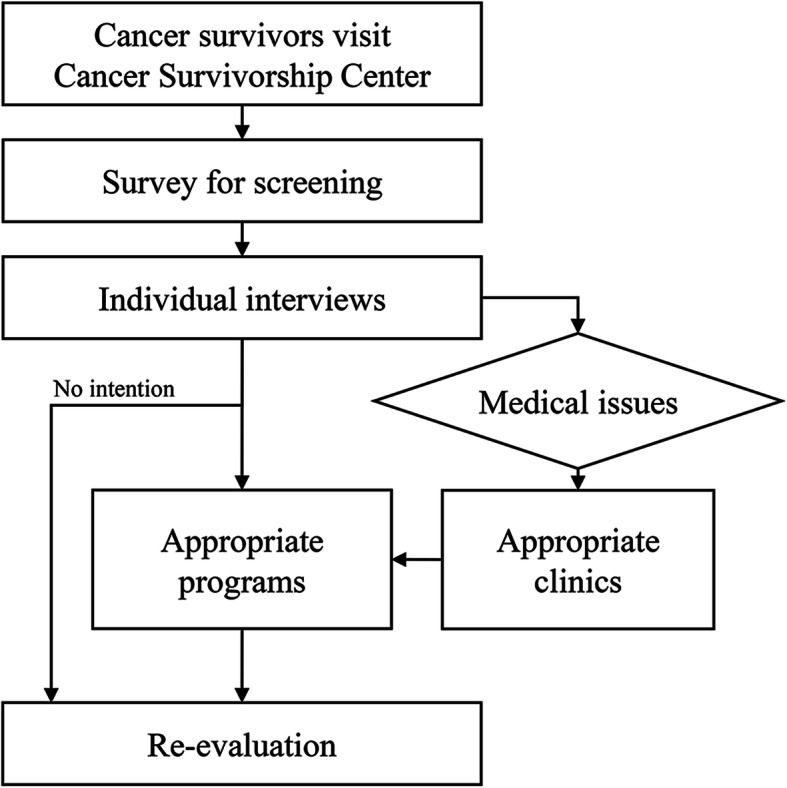
Flow chart of the process in the cancer survivorship program

After signing informed consent, enrolled cancer survivors filled out the surveys on quality of life (Supplementary File 1). Once each survivor’s survey answers were reviewed, an experienced nurse conducted individual interviews for about an hour where each patient was asked further detailed questions about subjects listed in the Survivorship Assessment (NCCN Guidelines Version 2016) [9]. Furthermore, the nurse interviewed the patients more specifically focused on the issues with at least the three worst scores in the surveys. During the interviews, the nurse transcribed the consultation details as a text form, which was used to identify each patient’s issues and ultimately match an appropriate program for each issue. Categories and process of cancer survivor support programs provided at the center and patients’ participation records are shown in Table 1 and Fig. 1. The center’s programs cover a wide range of topics such as nutrition, exercise, meditation, family care, and art therapy. The second round of the survey for evaluating the change of the scores was performed when the patients visited the hospital 1 month after the enrollment. If the patients could not visit the center at the time, the nurse tried the survey by the phone calls.

**Table 1:** The provided cancer survivorship programs and the number of participants (*N* = 322)

Categories	No. of patients		No. of cumulative participants	
	n	(%)	Total	Mean (range)
Clinic	66	(20.5)		
Physical programs	150	(46.6)	293	2.0 (1–9)
Nutrition	87	(27.0)	99	1.1 (1–3)
Exercise	105	(32.6)	167	1.6 (1–6)
Health care	25	(7.8)	27	1.1 (1–2)
Emotional programs	106	(32.9)	335	3.2 (1–17)
Distress	71	(22.0)	262	3.7 (1–16)
Sleep	5	(1.6)	5	1.0 (1)
Relaxation	55	(17.1)	68	1.2 (1–4)
Family / Social programs	53	(16.5)	69	1.3 (1–3)
Family	26	(8.1)	34	1.3 (1–2)
Job	1	(0.3)	1	1.0 (1)
Socioeconomy	30	(9.3)	34	1.1 (1–3)
Events	72	(22.4)	121	1.7 (1–4)
Camps	43	(13.4)	43	1.0 (1)
Others	60	(18.6)	78	1.3 (1–3)

### Sample size

A total of 561 patients initially signed up at our center between May 2018 and July 2019 to participate in the Korean Cancer Survivorship Pilot Project. The analysis excluded surveys filled out by ten patients as they did not thoroughly answer their questionnaires. Among those 551 patients, 322 patients completed the second round of surveys a month after the initial survey. To track changes in survey scores, we used survey sets from those 322 patients instead of those of 551. On the other hand, we used all of the individual interviews conducted on 551 patients for text analysis to ensure that machine learning had enough data to draw conclusions. Separate IRB approval for further analysis, including the text analysis, was obtained.

### Measurements and outcomes

The survey questions were designed to assess patients’ distress, fatigue, pain, insomnia, anxiety, depression based on the following tools: the National Comprehensive Cancer Network (NCCN) distress thermometer, a Korean version of the problem list and a five dimensional tool for visual analogue scales of anxiety, depression, fatigue, pain, and outcome domain of need for help [10, 11]. The score systems were scaled from 0 (representing not at all or in the best condition) to 10 (in the worst condition). Also, EuroQol Visual Analogue Scale (EQ-VAS) was measured, ranging from 0 to 100 [10–12]. These survey measurements were developed and validated in previous Korean studies [10–12]. The survey also asked for general information about survivorship such as disease status, cancer treatment, socioeconomic condition, lifestyle behaviors and their physical, psychological, and practical needs, and problems. “High distress” was defined with a score 4 or higher, commonly considered severe distress [13].

This study’s primary endpoint was the improvement in distress and fatigue scores at the time of the one-month follow-up. We grouped and compared the patients based on the improvement of the scores; the “improved” group indicated the patients had a higher score at follow-up visits, and the “not improved” group was defined by a follow-up score that was the same as or less than the initial score.

### Text analysis

Machine learning-based text analysis was conducted to identify the survivors’ hidden needs that the survey might not have captured. The text analysis process used Word2Vec, which is an advanced version of the neural network language model (NNLM) that provides a word embedding function by turning words into vectors [14]. Word2Vec uses previously mentioned words or context to predict ensuing words and content. Recognizing that Korean words are not separated by spaces like in English, the part of speech (POS) tagging was used to distinguish each individual word. The study specifically used the Python (version 3.4) package genism model from the Word2vec method and Konlpy for the POS tagging of Korean [15]. Once words are tokenized, we extracted nouns exclusively, which was followed by the post-processing stage extracted list was reviewed to add or delete additional words to the library. Afterwards, the nouns were extracted in the order of the keywords by using the Most_similar function of the gensim model. The dimensionality of the word vector was 300, the minimum frequency of appearance was 50, and the window size, which means the number of surrounding words, was 8. After the extraction of words, the top10 ranked words were selected according to the relative similarity scores of each keyword including Health, Stress, Fatigue, Pain. Insomnia, Anxiety, and Depression. To quantify the result, mentioning the selected words were checked in the text of the interview for each patient. The counts for 10 words related with each keyword such as Stress and Fatigue, by frequency of mentions, were included in the statistical analysis as well as the survey results.

A network map was made with nodes and edges to visualize the relationship among the words. The nodes meant each word and their diameters reflected the frequency of mentions in the interviews, and the weights of the edges showed the relative similarities between two words. As the most basic centrality analysis measure, the degree centrality was defined as the count of the number of unique edges connected to the node [16, 17]. This technique was used to determine the network’s central node by measuring the degree of the edge between the nodes constituting the network and other nodes directly connected to the network. In this study, the word with the highest degree centrality score implies the frequency along with other words during the interview. Thus, significant keywords can be identified through degree centrality analysis.

### Statistical analysis

Appropriate tests were selected for comparison of characteristics between the groups of high and low distress levels and univariable analysis for the improvement of the scores; Fisher’s exact test for discrete variables, and Mann Whitney U-test for continuous variables. For the comparison between the groups based on the improvement of scores, multivariable analysis was performed using the logistic regression including the variables at *p* < 0.1 from the univariable analysis. Values were considered statistically significant when *p* < 0.05. Statistical analyses were performed using R 3.6.2 (R Development Core Team, Vienna, Austria, http://www.R-project.org).

## Results

### Patient characteristics

Table 2 displays the characteristics for the 322 patients who performed the 1-month follow-up survey. The majority of the patients were young (< 50 years, 81.1%), female (79.5%), less than 6 months from the end of treatment (70.5%), diagnosed with breast cancer (86.0%), early stages (75.2%), and previously healthy patients without comorbidities (74.8%). Among these patients, almost 83% of the patients participated in the cancer survivor support programs or clinics (*n* = 266, 82.6%) as shown in Supplementary Table 1 (Supplementary File 2). Initial and follow-up mean scores for EQ-VAS, distress, fatigue, pain, insomnia, anxiety, and depression are shown at the bottom of Table 2. All scores showed improvement after 1 month. Improvement at the 1 month follow-up was noted in about 50% of the patients in all categories. In the initial survey (Supplementary Table 1 in Supplementary File 2), participants’ most frequently experienced physical problems were in exercise (33.2%), followed by difficulties in memory or concentration (25.5%), and in nutrition (24.5%). Among emotional problems, more than 30% of the patients expressed difficulties with sleep and worry. Practical problems were relatively less common than physical or emotional problems. As for needs, 50.9 and 44.7% of the patients sought help in nutrition and exercise, respectively, which was higher than the percentage of patients actually expressing physical problems with nutrition and exercise (24.5 and 33.2%, respectively). However, for sleep or emotional problems, only a part of the patients experiencing those problems expressed the need for help.

**Table 2:** Characteristics of the cancer survivors

Variables	Total *N* = 322	Distress < 4 *n* = 176	Distress ≥4 *n* = 146	*p*-value
Age, median (range)	50 (25–79)	51 (32–79)	49 (25–73)	0.043
< 50 years	261 (81.1%)	73 (41.5%)	78 (53.4%)	
≥ 50 years	61 (18.9%)	103 (58.5%)	68 (46.6%)	
Sex				0.418
Male	8 (2.5%)	6 (3.4%)	2 (1.4%)	
Female	314 (97.5%)	170 (96.6%)	144 (98.6%)	
Time from end of treatment				0.277
< 6 months	227 (70.5%)	129 (73.3%)	98 (67.1%)	
≥ 6 months	95 (29.5%)	47 (26.7%)	48 (32.9%)	
Cancer types				0.770
Breast cancer	277 (86.0%)	150 (85.2%)	127 (87.0%)	
Others	45 (14.0%)	26 (14.8%)	19 (13.0%)	
Cancer stages				0.646
Early (I - II)	242 (75.2%)	130 (73.9%)	112 (76.7%)	
Advanced (III - IV)	80 (24.8%)	46 (26.1%)	34 (23.3%)	
Op				0.764
Not done	11 (3.4%)	7 (4.0%)	4 (2.7%)	
Done	311 (96.6%)	196 (96.0%)	142 (97.3%)	
Chemotherapy				0.140
Not done	139 (43.2%)	83 (47.2%)	56 (38.4%)	
Done	183 (56.8%)	93 (52.8%)	90 (61.6%)	
Radiotherapy				0.533
Not done	39 (12.1%)	19 (10.8%)	20 (13.7%)	
Done	283 (87.9%)	157 (89.2%)	126 (86.3%)	
Other treatment				0.456
Not done	154 (47.8%)	88 (50.0%)	66 (45.2%)	
Done^a^	168 (52.2%)	88 (50.0%)	80 (54.8%)	
Comorbidities				0.566
No	241 (74.8%)	129 (73.3%)	112 (76.7%)	
Yes	81 (25.2%)	47 (26.7%)	34 (23.3%)	
Smoking				0.865
No	292 (90.7%)	161 (91.5%)	131 (89.7%)	
Past	22 (6.8%)	11 (6.2%)	11 (7.5%)	
Current	8 (2.5%)	4 (2.3%)	4 (2.7%)	
Drinking				0.368
No	299 (92.9%)	166 (94.3%)	133 (91.1%)	
Yes	23 (7.1%)	10 (5.7%)	13 (8.9%)	
Psychological counseling				0.012
No	284 (88.2%)	163 (92.6%)	121 (82.9%)	
Yes	38 (11.8%)	13 (7.4%)	25 (17.1%)	
Working				0.111
No	260 (80.7%)	136 (77.3%)	124 (84.9%)	
Yes	62 (19.3%)	40 (22.7%)	22 (15.1%)	
Married or living with a partner				0.161
No	257 (79.8%)	146 (83.0%)	111 (76.0%)	
Yes	65 (20.2%)	30 (17.0%)	35 (24.0%)	
Caregiver				< 0.001
None or non-family	60 (18.6%)	20 (11.4%)	40 (27.4%)	
Family member	262 (81.4%)	156 (88.6%)	106 (72.6%)	
Pregnancy plan				1.000
No	317 (98.4%)	173 (98.3%)	144 (98.6%)	
Yes	5 (1.6%)	3 (1.7%)	2 (1.4%)	
Living a long distance				0.023
No	304 (94.4%)	161 (91.5%)	143 (97.9%)	
Yes	18 (5.6%)	15 (8.5%)	2 (1.4%)	
Initial scores^b^				
EQ-VAS	64 ± 19	67 ± 19	59 ± 18	< 0.000
Distress	3.7 ± 2.5	1.7 ± 1.1	6.0 ± 1.6	< 0.000
Fatigue	4.4 ± 2.4	3.6 ± 2.1	5.5 ± 2.3	< 0.000
Pain	2.4 ± 2.3	1.9 ± 1.9	3.1 ± 2.5	< 0.000
Insomnia	3.1 ± 2.8	1.9 ± 2.0	4.6 ± 2.8	< 0.000
Anxiety	2.9 ± 2.7	1.3 ± 1.5	4.8 ± 2.7	< 0.000
Depression	2.8 ± 2.8	1.1 ± 1.5	4.8 ± 2.7	< 0.000
Scores at 1 month^b^				
EQ-VAS	74 ± 16	77 ± 14	70 ± 17	< 0.000
Distress	2.6 ± 1.8	2.1 ± 1.6	3.3 ± 1.8	< 0.000
Fatigue	3.3 ± 2.3	3.0 ± 2.3	3.8 ± 2.3	0.002
Pain	1.8 ± 2.1	1.6 ± 1.9	2.2 ± 2.2	< 0.000
Insomnia	1.9 ± 2.4	1.1 ± 1.7	2.9 ± 2.7	< 0.000
Anxiety	1.7 ± 1.9	0.9 ± 1.4	2.6 ± 2.1	< 0.000
Depression	1.6 ± 2.0	0.9 ± 1.4	2.6 ± 2.3	< 0.000
Number of improved patients				
EQ-VAS	197 (61.2%)	102 (58.0%)	95 (65.1%)	0.234
Distress	182 (56.5%)	58 (33.0%)	124 (84.9%)	< 0.000
Fatigue	188 (58.4%)	94 (53.4%)	94 (64.4%)	0.061
Pain	157 (48.8%)	79 (44.3%)	79 (54.1%)	0.101
Insomnia	168 (52.2%)	83 (47.2%)	85 (58.2%)	0.062
Anxiety	172 (53.4%)	72 (40.9%)	100 (68.5%)	< 0.000
Depression	165 (51.2%)	61 (34.7%)	104 (71.2%)	< 0.000

^a^Most of patients with other treatment had hormonal therapies for breast cancer except radio-frequency ablation for one patient with liver cancer. ^b^Continuous variables were represented by mean ± standard deviation

We checked the different characteristics between patients with high and low distress (Table 2). Compared to low distress patients (less than 4), patients with higher distress (4 or higher) were younger (*p* = 0.043), more previous counseling history (*p* = 0.012), and less family caregiver (*p* < 0.001). Though the one-month follow-up scores of the initially high distress group were still worse, they showed a greater improvement rate (the number of improved patients in all patients) in distress (84.9% versus 33.0%, p < 0.001), anxiety (68.5% versus 49.9%, p < 0.001), and depression (71.2% versus 34.7%, p < 0.001) at the one-month follow-up than the initially low distress patients.

### Text analysis for individual interviews

**Fig. 2:**
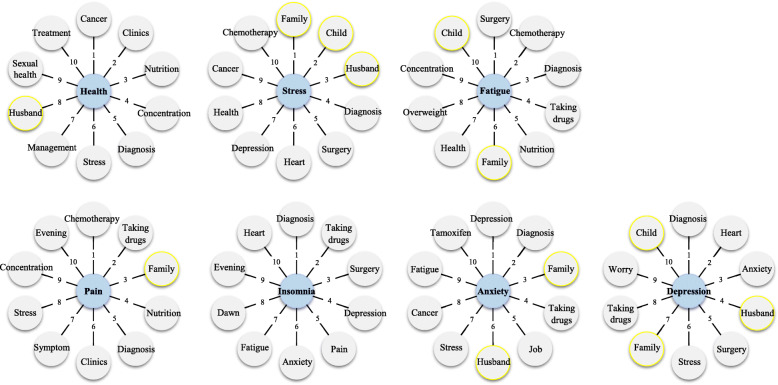
The keywords (blue nodes) and the top 10 ranked correlated words (gray nodes) from the text analysis with the individual interviews; Gray nodes with yellow boundary, family-related words; and the numbers, the ranks of words

The top 10 ranked vocabularies were selected by correlation with each keyword, Health, Stress, Fatigue, Pain, Insomnia, Anxiety, and Depression, from the text analysis (Fig. 2). Even after finishing cancer treatment, many of the words correlating with the keywords that the patients used during the interviews were therapeutic-related. Interestingly, family-related words such as family, child, and husband showed the highest correlation with the keyword Stress. Family-related words also correlated with Fatigue, Anxiety, and Depression. Emotional words were mostly related to Insomnia and Depression. In comparison, physical words were frequently associated with Fatigue.

During the interviews, the mean counts for mentioning the ten words related to Stress and Fatigue were 2.4 and 2.8, respectively. The counts were compared according to the improvement of distress and fatigue scores, and both were significantly different between the improved group and not-improved group (*p* < 0.001 and 0.036, respectively, Tables 3 and 4).

**Table 3:** Univariable analyses and multivariable logistic regression for improvement of distress

Characteristics	Not improved (*n* = 140)		Improved (*n* = 182)			Logistic regression		
	n	(%)	n	(%)	p-value	OR	95% CI	p-value
Age, median (range)					0.002			
< 50 years	52	(37.1)	99	(54.4)				
≥ 50 years	88	(62.9)	83	(45.6)				
Op					0.063	5.296	0.741–47.637	0.114
Not done	8	(5.7)	3	(1.6)				
Done	132	(94.3)	179	(98.4)				
Other treatment					0.025	1.716	0.918–3.246	0.093
Not done	77	(55.0)	77	(42.3)				
Done	63	(45.0)	105	(57.7)				
Psychological counseling					0.003	2.197	0.751–6.968	0.162
No	132	(94.3)	152	(83.5)				
Yes	8	(5.7)	30	(16.5)				
Living a long distance					0.051			
No	128	(91.4)	176	(96.7)				
Yes	12	(12)	6	(3.3)				
Physical problems								
Appearance					0.063			
No	128	(91.4)	153	(84.1)				
Yes	12	(8.6)	29	(15.9)				
Dyspepsia					0.007	3.192	1.416–7.570	0.006
No	122	(87.1)	136	(74.7)				
Yes	18	(12.9)	46	(25.3)				
Memory or concentration					0.028			
No	113	(80.7)	127	(69.8)				
Yes	27	(19.3)	55	(30.2)				
Nausea					0.077			
No	126	(90.0)	151	(83.0)				
Yes	14	(10.0)	31	(17.0)				
Nasal dryness or fullness					0.090			
No	131	(93.6)	159	(87.4)				
Yes	9	(6.4)	23	(12.6)				
Skin dryness or itching					0.089			
No	119	(85.0)	140	(76.9)				
Yes	21	(15.0)	42	(23.1)				
Numbness in extremities					0.049			
No	114	(81.4)	130	(71.4)				
Yes	26	(18.6)	52	(28.6)				
Difficulties in exercise					0.044			
No	102	(72.9)	113	(62.1)				
Yes	38	(27.1)	69	(37.9)				
Emotional problems								
Nervousness					0.013			
No	123	(87.9)	140	(76.9)				
Yes	17	(12.1)	42	(23.1)				
Sadness					< 0.001			
No	131	(93.6)	144	(79.1)				
Yes	9	(6.4)	38	(20.9)				
Worry					0.002			
No	108	(77.1)	110	(60.4)				
Yes	32	(22.9)	72	(39.6)				
Loss of interest in daily life					0.056			
No	126	(90.0)	150	(82.4)				
Yes	14	(10.0)	32	(17.6)				
Problems with sleep					< 0.001			
No	109	(77.9)	100	(54.9)				
Yes	31	(22.1)	82	(45.1)				
Anxiety					< 0.001			
No	121	(86.4)	115	(63.2)				
Yes	19	(13.6)	67	(36.8)				
Depression					< 0.001			
No	123	(87.9)	118	(64.8)				
Yes	17	(12.1)	64	(35.2)				
Practical problems								
Health of family members					0.050			
No	126	(90.7)	151	(83.0)				
Yes	13	(9.3)	31	(17.0)				
Childcare					0.002			
No	129	(92.1)	146	(80.2)				
Yes	11	(7.9)	36	(19.8)				
Housework					0.034			
No	120	(85.7)	138	(75.8)				
Yes	20	(14.3)	44	(24.2)				
Relationship w/ others in Family					< 0.001			
No	139	(99.3)	162	(89.0)				
Yes	1	(0.7)	20	(11.0)				
Needs								
Secondary cancer prevention					0.020			
No	98	(70.0)	104	(57.1)				
Yes	42	(30.0)	78	(42.9)				
Fatigue management					0.033			
No	116	(82.9)	132	(72.5)				
Yes	24	(17.1)	50	(27.5)				
Exercise					0.032			
No	87	(62.1)	91	(50.0)				
Yes	53	(37.9)	91	(50.0)				
Care for sleep disorder					0.003			
No	126	(90.0)	140	(79.9)				
Yes	14	(10.0)	40	(23.1)				
Care for anxiety					0.001			
No	123	(87.9)	132	(72.5)				
Yes	17	(12.1)	50	(27.5)				
Care for depression					0.001			
No	128	(91.4)	142	(78.0)				
Yes	12	(8.6)	40	(22.0)				
Initial scores								
EQ-VAS	66 ± 19		61 ± 19		0.017			
Distress	2.0 ± 1.8		5.0 ± 2.2		< 0.001	2.777	2.138–3.734	< 0.001
Fatigue	4.1 ± 2.4		4.7 ± 2.4		0.035	0.681	0.571–0.802	< 0.001
Pain	2.4 ± 2.3		2.5 ± 2.3		0.670			
Insomnia	2.0 ± 2.3		4.0 ± 2.8		< 0.001			
Anxiety	1.5 ± 2.0		4.0 ± 2.8		< 0.001	1.253	1.009–1.563	0.043
Depression	1.5 ± 2.2		3.7 ± 2.9		< 0.001	0.753	0.597–0.937	0.013
Intervention								
Family care program					0.012	3.420	1.000–13.813	0.063
No	135	(96.4)	161	(88.5)				
Yes	5	(3.6)	21	(11.5)				
Counts for 10 words	1.9 ± 2.0		2.8 ± 2.4		< 0.001			
Therapeutic-related words					0.142			
No	30	(21.4)	27	(14.8)				
Yes	27	(78.6)	155	(85.2)				
Family-related words								
No	111	(79.3)	125	(68.7)	0.042			
Yes	29	(20.1)	57	(31.3)

**Table 4:** Univariable analyses and multivariable logistic regression for improvement of fatigue

Characteristics	Not improved (*n* = 134)		Improved (*n* = 188)			Logistic regression		
	n	(%)	n	(%)	p-value	OR	95% CI	p-value
Age, median (range)					0.054			
< 50 years	54	(40.3)	97	(51.6)				
≥ 50 years	80	(59.7)	91	(48.4)				
Time from end of treatment					0.025	0.651	0.363–1.163	0.147
< 6 months	85	(63.4)	142	(75.5)				
≥ 6 months	49	(36.6)	46	(24.5)				
Living a long distance					0.091	0.339	0.100–1.001	0.056
No	123	(91.8)	181	(96.3)				
Yes	11	(8.2)	7	(3.7)				
Physical problems								
Numbness in extremities					0.088			
No	95	(70.9)	149	(79.3)				
Yes	39	(29.1)	39	(20.7)				
Practical problems								
Economy or finance					0.075	0.409	0.188–0.868	0.021
No	109	(81.3)	167	(88.8)				
Yes	25	(18.7)	21	(11.2)				
Transportation					0.086	0.107	0.005–0.833	0.061
No	129	(96.3)	187	(99.5)				
Yes	5	(3.7)	1	(0.5)				
Needs								
Rehabilitation					0.038	0.449	0.211–0.940	0.035
No	115	(85.8)	175	(93.1)				
Yes	19	(14.2)	13	(6.9)				
Care for adverse effects					0.022			
No	108	(80.6)	169	(89.9)				
Yes	26	(19.4)	19	(10.1)				
Initial scores								
EQ-VAS	64 ± 19		63 ± 19		0.637			
Distress	3.2 ± 2.4		4.0 ± 2.6		0.011			
Fatigue	3.3 ± 2.2		5.2 ± 2.2		< 0.001	1.517	1.342–1.734	< 0.001
Pain	2.3 ± 2.3		2.5 ± 2.3		0.402			
Insomnia	2.8 ± 2.6		3.3 ± 2.9		0.073			
Anxiety	2.4 ± 2.5		3.3 ± 2.8		0.006			
Depression	2.4 ± 2.6		3.0 ± 2.9		0.035			
Intervention								
Participation					0.019	0.491	0.271–0.876	0.017
No	30	(22.4)	66	(35.1)				
Yes	104	(77.6)	122	(64.9)				
Family care program					0.021	3.895	1.298–13.825	0.022
No	129	(96.3)	167	(88.8)				
Yes	22	(3.7)	21	(11.2)				
Counts for 10 words	2.5 ± 1.9		3.0 ± 2.2		0.036			
Therapeutic-related words					0.375			
No	27	(20.1)	30	(16.0)				
Yes	107	(79.9)	158	(84.0)				
Family-related words					0.003	1.983	1.067–3.783	0.033
No	110	(82.1)	126	(67.0)				
Yes	24	(17.9)	62	(33.3)				

**Fig. 3:**
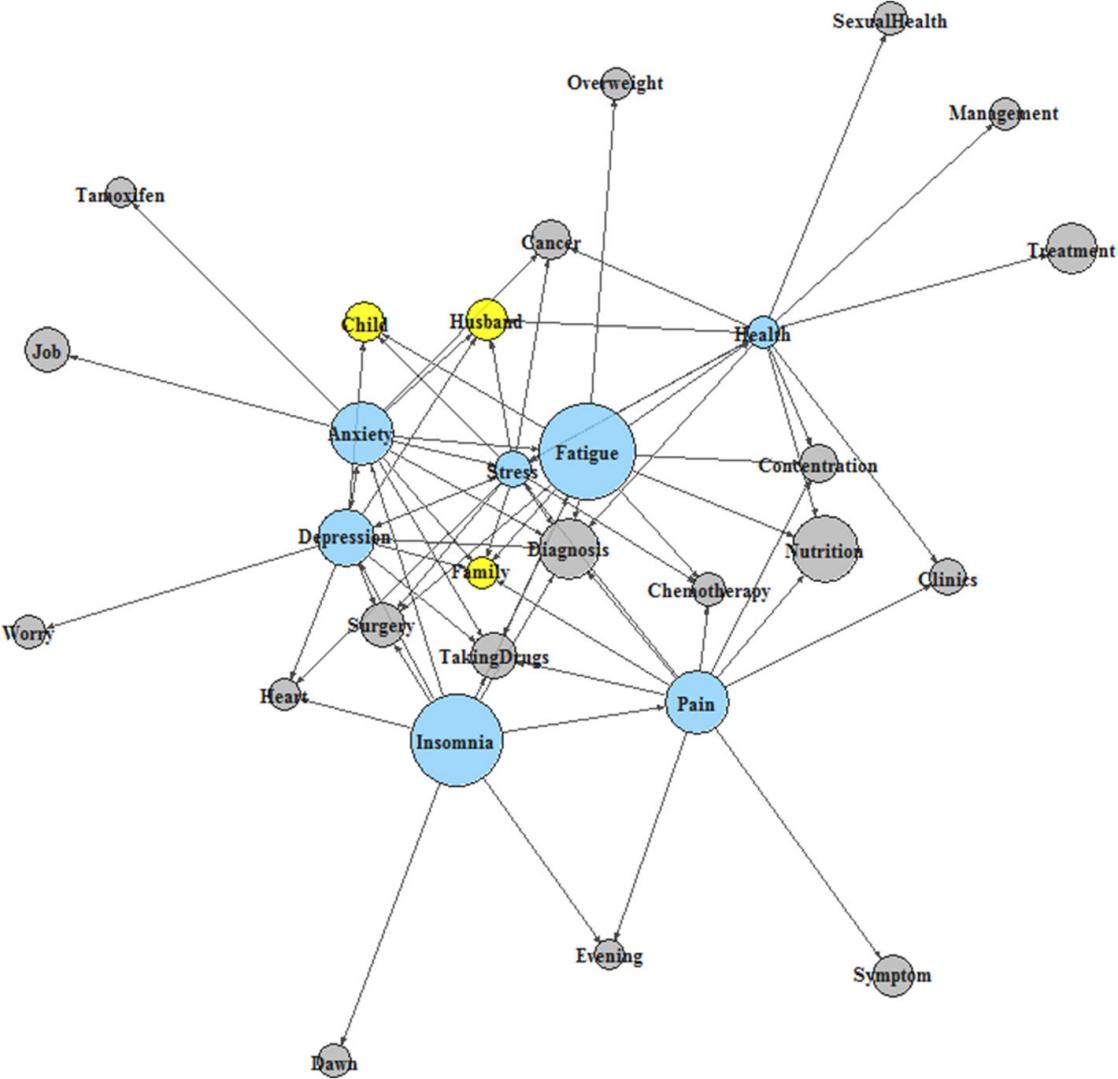
Networks among the keywords (blue nodes) and their related words (gray nodes, except yellow family-related words) from the text analysis with individual interviews; the size of each circle (node) was the frequency of the word mentioned in the interviews, and the length or weight of line (edge) between two circles were their relative correlation

The keyword network map for the seven keywords and the extracted words showed multiple rings linked with each other or spherical form in the center of the network and some branches (Fig. 3). It implied that the words had a very complex relationship. In the center, the keywords Fatigue and Stress were positioned, which were the main outcomes of this study. The degree centrality of the seven keywords was higher than other words (Supplementary File 2). Among all the words on the keyword network map, Fatigue and Stress recorded the highest centrality score, implying that these two keywords frequently mentioned and tightly connected with other words when the patients described their conditions during the interviews.

### Analysis of the scores

We performed uni- and multi-variable analysis with the characteristics, survey data, and the result of the text analysis. The significant factors of improvement in distress by the analyses are shown in Table 3. Multivariable analysis using logistic regression showed that dyspepsia (odds ratio (OR) 3.192, *p* = 0.006), the initial scores of distress (OR 2.777, *p* < 0.001), and anxiety (OR 1.253, *p* = 0.043) had a significantly positive relationship with the improvement in distress, and the initial score of fatigue (OR 0.681, p < 0.001), and depression (OR 0.753, *p* < 0.013) were negatively related to the improvement in distress.

Table 4 shows the results for the improvement in fatigue by the uni- and multivariable analysis. After adjusting other factors, initially higher scores of fatigue (OR 1.517, p < 0.001), participation in family care programs (OR 3.895, *p* = 0.022), and mention of family-related words during the interview (OR 1.983, *p* = 0.033) were significantly related to the improvement of fatigue. On the other hand, practical problems with economy or finance (OR 0.409, *p* = 0.021), need for rehabilitation (OR 0.440, *p* = 0.035), and participation in any clinic or other program in the center (OR 0.491, *p* = 0.017) were unfavorable factors for the improvement in fatigue.

## Discussion

Cancer survivors not only face cancer-related problems but also numerous physical, mental, and/or practical problems and challenges during and after cancer treatment. Subsequently, the needs of cancer survivors are frequently unmet during life after treatment. Although previous studies have addressed and tried to improve these issues, no effective solutions have been reported [18–20]. This could be due to the complexity of the problems that survivors experience and the various background and histories of individuals. Thus, the solutions from the previous studies using simple survey data may be insufficient to apply to a real-world situation. In this study, we used the latest technique, keyword network mapping using deep learning, and the conventional statistical analysis to better understand each individual cancer survivor with the survey and text review from the individual interviews. The main endpoints of this study were distress and fatigue, which are generally the most common chief complaints that in clinic patients express. Interestingly, distress and fatigue were also the most essential words in the keyword network map with the highest centrality scores. Statistical analysis showed that patients who participated in the family care program and those who used family-related words during the interview were significantly associated with fatigue improvement.

Distress, one of the main endpoints in this study, is recommended as one of the main indicators for managing cancer survivors in the NCCN guidelines [6, 13]. High emotional distress has been reported with a wide range from 9 to 50% [21–23], and our result was comparable. Since many patients were enrolled directly after finishing treatment, it may be seen that the psychological stress was mainly affected by the cancer treatment [24–26]. Additionally, the patients tended to focus on more physical or practical issues, not mental problems or stress issues, and high distress patients were younger and stayed without a family caregiver in our study. Though distress can be an overall indicator, its score may not show the detailed individual factors and its relationships. Furthermore, a recent report suggested that it was irrational to identify the cancer survivors’ condition by distress level alone [27]. An interesting point in our study was that dyspepsia was one of the factors related to distress; it may have been due to the residual side effects after radiotherapy to left-sided breast or systemic therapy. Alternatively, the possibility of somatization disorder can also be considered. Although it is not currently used as a psychiatric diagnosis, one of the previous diagnoses in DSM-IV names called “Hwabyung” was used as a characteristic psychosis code in Koreans [28]. The diagnosis of Hwabyeong included ambiguous physical symptoms such as dyspepsia while showing anxiety or depression [28]. The cohort of this study included many middle-aged Korean housewives who were susceptible to Hwabyeong. In Korea, many Koreans who have less tendency to express themselves psychiatry-wise reflect their issues by the vague physical symptoms.

Fatigue associated with cancer or cancer treatment, another main outcome of this study, requires some time to be recovered after treatment [3, 29]. However, long-term fatigue in cancer survivors is also majorly affected by factors other than cancer-related factors [30]. Many studies reported little association between persistent fatigue in cancer survivors and cancer treatment [31]. Thus, fatigue that persists after treatment may have different individual causes in aspects of lifestyles, practical situations, or even personalities [32]. Similarly, it is understandable that fatigue might not be improved if there were practical difficulties such as financial problems or lack of transportation [33]. For this reason, it is imperative that the cancer survivorship care team considers other various social or medical conditions of the patient such as physical recovery, moving distance, or family situation. Correspondingly, some reports suggest that individual life care in the community where the patients live or even private tele-cares through telephone or the Internet may be needed for effective personal supports [34, 35].

In our study, like distress, the improvement of fatigue was positively related to few factors. Interestingly, the participation of any programs or clinics was not a good factor for fatigue improvement. We believe that it was not a worse factor, but most of the patients were enrolled just after the end of treatments, and some patients needed more time to rest. Although practical problems of economy or finance and the need for rehabilitation were negatively associated with improvement in fatigue, participating in the family care program was a positive factor for fatigue improvement. The family care program was not education for the family members as caregivers, as family members of cancer survivors. The program’s purpose was to help the patients adapt to their role in the family after cancer treatment. In this study, most of the patients were diagnosed with breast cancer, and characteristics of the breast cancer population in Korea is that the median age is younger compared to the Western population, with many breast cancer patients younger than 50 years old. Accordingly, our results may reflect the situations of many young female cancer patients in which they need to maintain their lives as the caregivers for other family members such as their babies, husband, or older parents, rather than expressing their need to their family and society [36]. Even after finishing cancer treatment, their expected role and performance in the family may be the same as before their diagnosis. This could stem from the cultural notion of the close and united relationships as a family being one of the most critical values rather than a separate individual in Korea. Therefore, it is important to note that some cancer survivors need thoughtful considerations and programs for their role in their family and community.

Our study’s uniqueness is that we used a new approach for analysis through text analysis. At the time of registration, each participant was interviewed individually by a nurse, trained for this project. Because time or budget limitations, few other centers could not interview all cancer survivors in depth. Even if the interviews were performed in our institution, it was a challenge to find an appropriate method to analyze the text data. Recently in Korea, analysis techniques for natural languages or free text analysis were developed [15], and have been used widely in non-medical parts such as marketing. However, the studies in medicine related to text analysis are scarce, and no significant results have yet been reported [37]. Although many researchers have tried to work with the electric health records (EMR) or similar systems in the hospitals, limitations existed due to the rigid structure of EMR and its information security issue. Our dataset included simple and large sets of interview results in text form as well as the survey form from the project, and we were able to perform text analysis without considering the limitations arising from the structure of EMR or security issues.

In the survey, patients were asked to answer questionnaires for each category of distress, fatigue, pain, insomnia, anxiety, depression, and EQ-VAS. However, there were very complex links in the text analysis that were difficult to interpret among the keywords in the interviews. Using the keyword network map, we showed the relationship among the words, which was previously hard to show using the survey results. In the keyword network map, each keyword showed a different frequency and centrality, and the keywords at the center with the highest centrality were fatigue and distress. Additional text analysis showing the top 10 words correlated with the keywords (health, stress, fatigue, pain, insomnia, anxiety and depression) (Fig. 2) indicated that there were still many treatment-related statements even after finishing treatment. Considering that the patients were enrolled directly after finishing the treatment, it seemed that the patients were still affected by cancer at the survey and interview time [36]. In severe cases, some patients may be affected by cancer, like a trauma that may last for a long time and need psychiatric interventions [38]. Therefore, further emphasis of post-treatment care and support for cancer survivors is required. Another finding in this additional text analysis was that the family-related vocabularies such as children and husband were important words related to stress, anxiety, and depression. This may be due to the concerns that the patients feel responsible about taking care of other family members. In addition, multivariate analysis showed that family-related vocabulary and participation in the family care program were related to fatigue improvement. Although only 15% of the patients expressed difficulties for child rearing in the initial survey, which may lead to no statistical significance, text analysis was able to catch the importance of issues related to family for cancer survivors. Thus, it should be noted that individual interviews are important to grasp the complex issues of that are not comprehensible through simple survey. To provide practical support to cancer survivors, a simple follow-up with indicators such as distress level or short clinic sessions may not be sufficient. Therefore, our institution has been carrying out surveys and rather lengthy interviews whenever possible and when necessary to help more practical problems of each patient. As this study confirmed that the family care program helped to improve fatigue, we will continue to take further individual actions.

There were some limitations in this study. First, the population of the study had a selection bias. We used the existing data with the registered patients in the project, making it impossible to compare to other groups such as non-registered patients or other specific disease sites. Additionally, due to the limitations in human resources and budget, individual interviews for all patients in real-world situation might be difficult. In the future, personalized care in our cancer survivorship center will be established with criteria for an effective operation to fully support those who are in need. Finally, the deep learning techniques and analytic methods for the text analysis were relatively limited due to the small number of patients.

## Conclusions

Our cancer survivorship project showed favorable results in most aspects, including distress and fatigue. Particularly, considering the large portion of young breast cancer survivors and Korean culture, the participation in the family care programs was effective for improving fatigue. Moreover, text analysis with deep learning to use the individual interviews showed that the mention of family-related words during interviews was associated with fatigue improvement. It implied that the interviews had positive effects on the patients to participate in the family care program, which consequently lead to improvement in fatigue. This study will help health professionals provide more effective and personalized approaches to cancer survivorship.

## Supplementary Information

**Additional file 1. **The questions for the survey.

**Additional file 2: Supplementary Table 1**. Survey results (*N* = 332). **Supplementary Table 2**. Degree centrality in the network with the extracted words.

## Data Availability

Sharing the datasets generated and/or analyzed in the current study is not available due to patient-identifiable privacy information.

## Abbreviations

NCCN: National Comprehensive Cancer Network
EQ-VAS: EuroQol Visual Analogue Scale
NNLM: Neural network language model
POS: Part of speech
OR: Odds ratio
EMR: Electric health records
