# Supplementary material for: Factors to improve distress and fatigue in Cancer survivorship; further understanding through text analysis of interviews by machine learning
Source: BMC Cancer. 2021 Jun 27;21:741. doi: 10.1186/s12885-021-08438-8 (PMC8237475; doi:10.1186/s12885-021-08438-8)
Supplement: Supplementary file 1 — Additional file 1. The questions for the survey. [file 12885_2021_8438_MOESM1_ESM.docx]

**Notice: This document was translated in English for the purpose of the report. The actual version of the survey was written in Korean language for the Korean patients. Although it includes all contents of the survey, it can have some translational differences from the actual Korean version of the survey form.**

| **Registration no.** |  | **Name** |  | **Sex** | **M / F** | **Date** | **. .** |
| --- | --- | --- | --- | --- | --- | --- | --- |

**1. How to participate in the cancer survivorship program?**

① Recommended by the doctor for cancer treatment.

② Recommended by other hospital staffs (nurses, physical therapists, etc.)

③ Recommended by other patients.

④ Recommended by family members or friends.

⑤ In-hospital advertisements (posters, leaflets, banners, etc.)

⑥ Media (newspaper, broadcasting, radio, television, etc.)

⑦ Others ( )

**2. Please check (☑) the screening test which you performed previously and when it was in the following table.**

| **Cancer type** | | **Screening test** | **The latest test** | | | | | | |
| --- | --- | --- | --- | --- | --- | --- | --- | --- | --- |
|  |  |  | I don’t know | Never done | Within 1 year | 1 ~ 2 years ago | 2 ~ 5 years ago | 5 ~ 10 years ago | > 10 years |
| 1 | Stomach cancer | Gastroscopy | □ | □ | □ | □ | □ | □ | □ |
|  |  | GI series | □ | □ | □ | □ | □ | □ | □ |
| 2 | Colorectal cancer | Stool occult blood | □ | □ | □ | □ | □ | □ | □ |
|  |  | Colonoscopy | □ | □ | □ | □ | □ | □ | □ |
| 3 | Liver cancer | Ultrasonography | □ | □ | □ | □ | □ | □ | □ |
|  |  | AFP | □ | □ | □ | □ | □ | □ | □ |
| 4 | Breast cancer | Mammography | □ | □ | □ | □ | □ | □ | □ |
|  |  | Exam by a doctor or yourself | □ | □ | □ | □ | □ | □ | □ |
| 5 | Uterine cervical cancer | Pap smear | □ | □ | □ | □ | □ | □ | □ |

* Only for the patients with Cirrhosis, hepatitis B antigen positive, hepatitis C antibody positive, chronic liver disease caused by hepatitis B or C virus.

3. **Physical problems; please check (☑) any difficulties you have experienced in the past week.**

| 1. Appearance | □ | 11. Indigestion | | □ |
| --- | --- | --- | --- | --- |
| 2. Bathing or dressing | □ | 12. Swallowing difficulty | | □ |
| 3. Breathing | □ | 13. Difficult in memory or focus | | □ |
| 4. Difficulty in urination | □ | 14. Oral ulcer | | □ |
| 5. Constipation | □ | 15. Nausea | | □ |
| 6. Diarrhea | □ | 16. Dryness or obstruction of nose | | □ |
| 7. Edema | □ | 17. Dry skin or itch | | □ |
| 8. Fever | □ | 18. Alcohol or drug abuse | | □ |
| 9. Discomfort to move | □ | 19. Numbness in hands or feet | | □ |
| 10. Loss of appetite | □ | 20. Others ( ) | |  |
| 21. Have you had any nutritional difficulties during the past week? | | | □ Yes □ No | |
| 21-1. Weight within the past 3 months | | | □Increase □Decrease □No change | |
| 21-2. Oral intake within the past 3 months | | | □Increase □Decrease □No change | |
| 21-3. Do you know guidelines for nutrition supports for the cancer patients? | | | □ Yes □ No | |
| 22. Have you had any trouble with fatigue during the past week? | | | □ Yes □ No | |
| 22-1. How severe have you felt fatigued? | | | 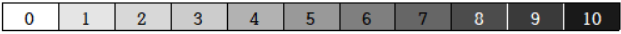None Extremely severe | |
| 23. Have you had any trouble with pain during the past week? | | | □ Yes □ No | |
| 23-1. How severe have you felt the pain? | | | None Extremely severe 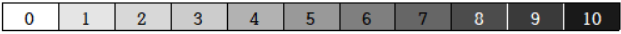 | |
| 24. Have you had any trouble with physical exercise during the past week? | | | □ Yes □ No | |
| 24-1. Do you do physical exercise regularly? | | | □ Yes □ No | |
| 24-2. If not, why? Please check the followings or specify. | | | □ Others ( ) | |
| □ Due to no time to do □ Due to physical symptoms such as pain □ I don’t know how to exercise | | | | |
| 24-3 Any symptom related to edema | | | □ Yes □ No | |
| 25. Have you had any trouble with sexual health during the past week? | | | □ Yes □ No | |
| 25-1. Have you had any trouble with sexual health since the cancer diagnosis? | | | □ Yes □ No | |
| 26. EQ-VAS: How is your health condition today? Please evaluate it from 0 (no problem or best) to 100 (extremely severe or worst)? | | | _____________ point | |

**4. Emotional problems; please answer or check (☑) any difficulties you have experienced in the past week.**

4-1. Distress thermometer: how much has been the level of your distress during the past week?


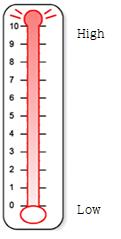


__________ point

4-2. Please check (☑) any difficulties you have experienced during the past week.

| 1. Annoying | □ | 5. Sleep (Insomnia or excessive sleep) | | □ |
| --- | --- | --- | --- | --- |
| 2. Sadness | □ | 6. Anxiety | | □ |
| 3. Worry | □ | 7. Depression | | □ |
| 4. loss of interest in daily activities | □ |  | | □ |
| 5-1. How severely have your insomnia (hypersomnia) been during the past week? | | | None Extremely severe 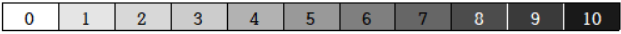 | |
| 6-1. How anxious have you been during the past week? | | | None Extremely severe 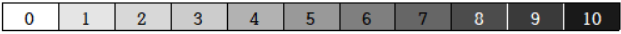 | |
| 7-1. How depressed have you been during the past week? | | | None Extremely severe 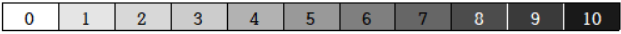 | |

**5. Other problems: please answer or check (☑) any difficulties you have experienced in the past week.**

| 1. Economic or financial problem | □ | 7. Relationship with your child (children) | □ |
| --- | --- | --- | --- |
| 2. Transportation | □ | 8. Relationship with your spouse | □ |
| 3. Work or school | □ | 9. Relationship with other family members | □ |
| 4. Family health | □ | 10. Pregnancy | □ |
| 5. Childcare | □ | 11. Religious or spiritual problem | □ |
| 6. Housework | □ |  |  |

**6. Needs for any help (consult or information)**

| 1. Information on prevention or screening for the secondary cancer | □ | 9. Social Welfare | □ |
| --- | --- | --- | --- |
| 2. Vaccination | □ | 10. Sleep problem | □ |
| 3. Nutrition or foods | □ | 11. Anxiety | □ |
| 4. Fatigue | □ | 12. Depression | □ |
| 5. Pain | □ | 13. Treatment complication | □ |
| 6. Workout | □ | 14. Quitting Smoking | □ |
| 7. Rehabilitation | □ | 15. Others ( ) | □ |
| 8. Sexual health | □ |  |  |

**7. Personal and practical information**

| 7-1. Do you work now? | □ Yes □ No |
| --- | --- |
| 7-2. Marriage | □Single □Married or living with a partner |
| 7-3. Caregiver(s) | □Spouse □Parents □Son/daughter(s)  □Brother/sister(s) □None (self-care) □ Friend(s) |
| 7-4. Smoking | □ Never □ Ex-smoker □ Current smoker |
| 7-5. Alcohol | □ Yes □ No |
| 7-6. Plan for pregnancy | □ Yes □ No |
