# Supplementary material for: Factors to improve distress and fatigue in Cancer survivorship; further understanding through text analysis of interviews by machine learning
Source: BMC Cancer. 2021 Jun 27;21:741. doi: 10.1186/s12885-021-08438-8 (PMC8237475; doi:10.1186/s12885-021-08438-8)
Supplement: Supplementary file 2 — Additional file 2: Supplementary Table 1. Survey results (N = 332). Supplementary Table 2. Degree centrality in the network with the extracted words. [file 12885_2021_8438_MOESM2_ESM.docx]

Supplementary Table 1. Survey results (N = 332)

| Questionnaires | Total | |
| --- | --- | --- |
|  | n | (%) |
| Physical problems  Appearance  Bathing or dressing  Breathing  Urinary change  Constipation  Diarrhea  Swelling or edema  Heating sense or fever  Inconvenience in activities  Loss of appetite  Dyspepsia  Dysphagia  Memory or concentration  Oral ulcer  Nausea  Nasal dryness or fullness  Skin dryness or itching  Alcohol or drug overuse  Numbness in extremities  Weight change  On exercise  Difficulties in exercise  Difficulties in nutrition  Difficulties in sexual activities | 41  39  20  27  41  20  71  43  32  50  64  1  82  9  45  32  63  0  78  140  217  107  79  65 | (12.7)  (12.1)  (6.2)  (8.4)  (12.7)  (6.2)  (22.0)  (13.4)  (9.9)  (15.5)  (19.9)  (0.3)  (25.5)  (2.8)  (14.0)  (9.9)  (19.6)  -  (24.2)  (43.5)  (67.4)  (33.2)  (24.5)  (20.2) |
| Emotional problems  Nervousness  Sadness  Worry  Loss of interest in daily life  Problems with sleep  Anxiety  Depression | 59  47  104  46  113  86  81 | (18.3)  (14.6)  (32.3)  (14.3)  (35.1)  (26.7)  (25.2) |
| Practical problems  Economy or finance  Transportation  Work / School  Health of family members  Childcare  Housework  Relationship w/ children  Relationship w/ spouse  Relationship w/ others in family  Pregnancy potential  Spiritual or religious issues | 46  6  23  44  47  64  55  40  21  4  6 | (14.3)  (1.9)  (7.1)  (13.7)  (14.6)  (19.9)  (17.1)  (12.4)  (6.5)  (1.2)  (1.9) |
| Needs  Secondary cancer prevention  Vaccination  Nutrition  Fatigue management  Pain control  Exercise  Rehabilitation  Sexual health  Socioeconomical help  Care for sleep disorder  Care for anxiety  Care for depression  Care for adverse effects  Smoking clinic | 120  46  164  74  35  144  32  20  31  56  67  52  45  2 | (37.3)  (14.3)  (50.9)  (23.0)  (10.9)  (44.7)  (9.9)  (6.2)  (9.6)  (17.4)  (20.8)  (16.1)  (14.0)  (0.6) |

Supplementary Table 2. Degree centrality in the network with the extracted words

|  | degree centrality |
| --- | --- |
| Stress | 11.99895233 |
| Fatigue | 11.99888384 |
| Depression | 10.99903983 |
| Health | 10.99903762 |
| Pain | 10.99897653 |
| Anxiety | 10.99895364 |
| Insomnia | 9.998938262 |
| Diagnosis | 6.999396383 |
| Family | 4.999564647 |
| Taking Drugs | 4.999554336 |
| Husband | 3.999648571 |
| Surgery | 3.999644577 |
| Anti-Cancer | 2.999740183 |
| Nutrition | 2.999739587 |
| Cancer | 2.999739051 |
| Child | 2.999726951 |
| Concentration | 2.999719561 |
| Heart | 2.999716997 |
| Clinics | 1.999830186 |
| Evening | 1.999787151 |
| Management | 0.999909103 |
| Worry | 0.999908984 |
| Sexual Health | 0.99990648 |
| Therapy | 0.999906421 |
| Job | 0.999906182 |
| Symptom | 0.999905109 |
| Overweight | 0.99990505 |
| Tamoxifen | 0.99989903 |
| Dawn | 0.999885321 |
